# The many ways to age for a single yeast cell

**DOI:** 10.1002/yea.3020

**Published:** 2014-06-13

**Authors:** Didac Carmona-Gutierrez, Sabrina Büttner

**Affiliations:** Institute of Molecular Biosciences, University of GrazAustria

**Keywords:** programmed cell death, apoptosis, necrosis, ageing, replicative lifespan, chronological lifespan, yeast, altruism, adaptation, colony differentiation

## Abstract

The identification and characterization of the molecular determinants governing ageing represents the key to counteracting age-related diseases and eventually prolonging our health span. A large number of fundamental insights into the ageing process have been provided by research into the budding yeast *Saccharomyces cerevisiae*, which couples a wide array of technical advantages with a high degree of genetic, proteomic and mechanistic conservation. Indeed, this unicellular organism harbours regulatory pathways, such as those related to programmed cell death or nutrient signalling, that are crucial for ageing control and are reminiscent of other eukaryotes, including mammals. Here, we summarize and discuss three different paradigms of yeast ageing: replicative, chronological and colony ageing. We address their physiological relevance as well as the specific and common characteristics and regulators involved, providing an overview of the network underlying ageing in one of the most important eukaryotic model organisms.

## Introduction

The observation that the budding yeast *Saccharomyces cerevisiae* undergoes programmed cell death (PCD) with characteristic apoptotic changes initiated a new and exciting research field (Madeo *et al*., 1997). To date, the existence of regulated modes of cell death has been described in various microbial organisms, including bacteria, parasites such as *Leishmania* and *Trypanosoma*, or the slime mould *Dictyostelium discoideum*, and a plethora of molecular pathways as well as endogenous and exogenous stimuli of PCD induction have been identified. In addition, distinct types of unicellular PCD, first and foremost apoptosis and programmed necrosis, have been identified and characterized (Carmona-Gutierrez *et al*., 2010). Importantly, programmed necrosis shows a similar phenotype as ‘classical necrosis’ but differs in that it is largely executed in a coordinated fashion following an active regulatory mechanism and is not an uncontrolled process.

However, while multicellularity clearly benefits from, and even requires, controlled cellular demise to ensure normal development, tissue homeostasis and regulation and function of the immune system, the reason for the existence of PCD in unicellular organisms seems paradoxal at first, since death provides no advantage for an individual cell. If so, though, natural selection should have worked against the capability of unicellular organisms to undergo programmed suicide. Nevertheless, this trait was sustained throughout evolution, indicating an integral benefit even for single-celled species. In this case, it appears to serve the survival of a clonal population and raises the concept of ‘altruistic’ unicellular behaviour. As microbial life in natural habitats mainly occurs in clustered, social communities such as colonies and biofilms, which enable the cells to cope with ecological stresses and to efficiently adapt to a constantly changing environment, the death of an individual cell might be subsidiary to the survival of the ‘multicellular’ community. For *S. cerevisiae*, several physiological scenarios in which unicellular death seems to serve the survival of the clonal population have been described during the last 15 years, ranging from reproduction and development to interspecies competition and ageing. For instance, the rearrangement of the genome by meiosis (diploid cells) or mating (haploid cells) guarantees genetic variation and consequently provides an adaptive advantage to the population. Upon failure of either one of these processes, triggering PCD cleans the population from infertile or damaged cells, thus ensuring fitness and survival. In this line, haploid cells undergo PCD upon long-term exposure to pheromones without subsequent mating (Severin and Hyman, 2002; Zhang *et al*., 2006). Moreover, during meiosis of diploids to generate long-lived spores, a fraction of cells undergoes apoptosis (Knorre *et al*., 2005). A recent study could link the control of spore numbers to apoptotic processes, introducing the phenomenon of ‘programmed nuclear destruction’ (Eastwood *et al*., 2012). Under nutrient limitation to enforce spore number control, uncellularized meiotic products are actively degraded in a way that requires apoptotic DNA fragmentation (via the yeast endonuclease G *NUC1*) and an unusual form of autophagy that resembles PCD-associated mega-autophagy in plants. Besides these roles during sexual and asexual reproduction, additional physiological scenarios associated with PCD in yeast have been described, among which ageing has drawn most attention in the course of the last decade. Mostly, ageing is defined as the progressive loss of function in all constituents of living cells, leading to a decrease in both survival rate and reproductive capability. Several theories have been developed to explain the complex process of ageing, all of which are partly interrelated and mainly cluster into two categories, namely (a) the damage or error theories and (b) the programmed theories. The first group of theories, such as the ‘free radical theory’ or the ‘somatic DNA damage theory’, assumes environmental insults and subsequent accumulating damage as causative for ageing. In contrast, programmed theories, such as the ‘programmed longevity theory’, assume that ageing follows a biological clock and that lifespan and death are genetically programmed. To date, no consensus exists on the issue why organisms age.

**Figure 1:**
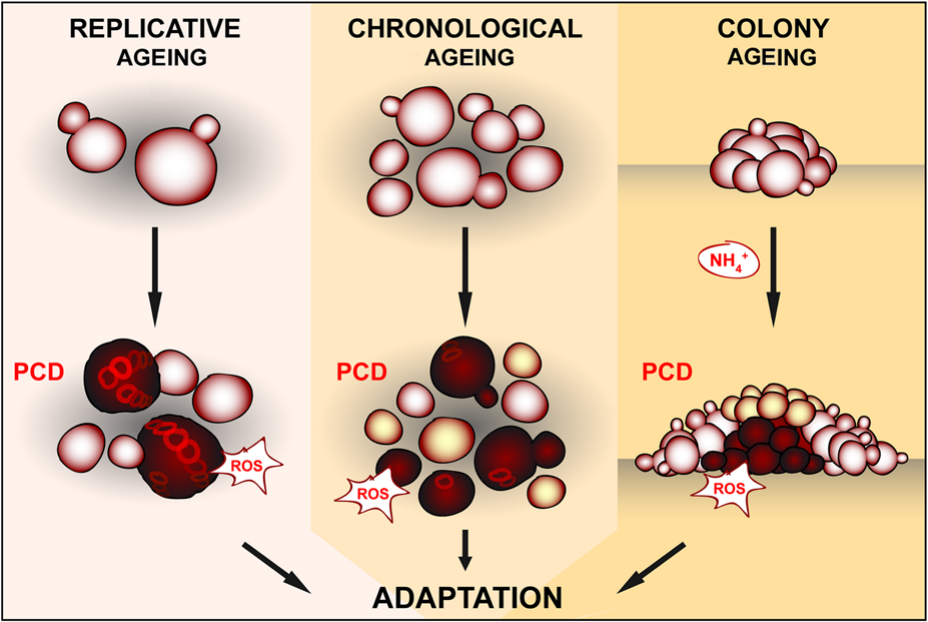
The three paradigms of yeast ageing: replicative, chronological and colony ageing. Programmed cell death (PCD) of replicatively old mother cells, chronologically aged cells and cells in the colony centre is accompanied by overproduction of reactive oxygen species (ROS). This death of individual cells ensures adaptation to the changing environment and long-term survival of the clonal population

Yeast has become the leading model organism for the study of genetic and mechanistic factors that affect human ageing and disease. The pathways and downstream factors identified to govern yeast senescence seem to be conserved to a large degree in higher eukaryotes and have been instrumental in deciphering ageing modulators in major model organisms up to the mouse (Longo *et al*., 2012). Here, we summarize and discuss current knowledge about the role and regulation of PCD during different forms of yeast ageing, including replicative, chronological and colony ageing. Besides their importance for microbial senescence and the resulting biotechnological, pathogenic and health-related implications, they also represent alternative paradigms to model mitotic and postmitotic ageing scenarios in higher eukaryotes (Figure 1). In addition, we also address – besides their specific characteristics – common denominators and putative mechanistic crossroads between the distinct ageing subtypes.

## Ageing in the mother cell: asymmetry and replicative lifespan

The replicative lifespan determines the number of divisions an individual mother cell undergoes before demise. This death results from the accumulation of damaged cellular material, which is asymmetrically segregated after each division between the daughter cell, which obtains the undamaged material, and the mother cell. In the wild, this contributes to the fast mitotic growth of a population upon nutrient-rich conditions while concurrently maintaining its fitness. Thus, daughters exhibit an intact replicative capacity to promote the population's survival at the cost of the mother cells, which increasingly lose their individual replicative potential. After typically 25–35 division cycles, replicatively aged mother cells start to die, showing apoptotic markers such as overproduction of reactive oxygen species (ROS), phosphatidylserine externalization and DNA fragmentation (Laun *et al*., 2001). Thus, replicative ageing mirrors senescence under proliferative conditions and may also model the ageing process of mitotically active cells in higher eukaryotes, including human stem cell populations. Notably, in recent years technically more refined and automated methods have been developed to remove daughter cells while retaining mother cells (Ryley and Pereira-Smith, 2006; Zhang *et al*., 2012). Such automatic approaches, for instance using microfluidic devices, overcome the difficulties posed by the arduous and time-consuming technique of classical manual micromanipulation. This allows the previously unfeasible accomplishment of large-scale screens and molecular phenotyping throughout the mother cell's lifespan.

Proteins are among the material asymmetrically distributed after division, resulting in the accumulation of damaged proteins in mother cells with each cycle. Accordingly, reduction of such damaged proteins seems to counteract the ageing process, since increased proteasome activity via activation of the transcription factor Rpn4p promotes replicative lifespan (Kruegel *et al*., 2011). Nevertheless, the modulation of replicative ageing through proteasome activity – which seems to act independently from the Sir2p- and nutrient-sensing pathways (see below) – needs further investigation. Importantly, mitochondria are also asymmetrically distributed upon cell division (McFaline-Figueroa *et al*., 2011) and may thus be a driving lethal factor during replicative ageing. Detrimental ROS are mainly produced in damaged mitochondria and, although the causal connection between oxidative damage and replicative ageing needs further clarification, both mitochondrial fragmentation and ROS production already start increasing after a few divisions (Lam *et al*., 2011). Although mitochondria play a determinant role in regulating the activation of resident pro-apoptotic factors, such as the endonuclease G-homologue Nuc1p, the apoptosis-inducing factor Aif1p or the NADH dehydrogenase Ndi1p (Büttner *et al*., 2007; Wissing *et al*., 2004; Li *et al*., 2006), the effect of these proteins on apoptotic death during replicative ageing still needs to be determined. However, the lack of the yeast BH3-only protein Ybh3p, the youth protein Uth1p or the yeast orthologue of the translationally controlled tumour protein Mmi1p (Büttner *et al*., 2011; Rinnerthaler *et al*., 2006; Kennedy *et al*., 1995), all connected to the mitochondrial pathway of apoptosis, causes an increase of replicative lifespan, indicating that distinct components of the (mitochondrial) apoptotic machinery are indeed involved in the determination of replicative lifespan. As these proteins have been described to have pro-apoptotic (Ybh3p, Uth1p) or anti-apoptotic (Mmi1p) roles, respectively, and in addition have been reported to function in other cellular processes besides cell death, the exact relationship between apoptosis and replicative ageing in general, and the mechanisms by which mitochondria impact on replicative ageing in particular, remain to be further elucidated. However, it seems clear that a modulatory mitochondrial–nuclear axis is critical; replicative lifespan can be increased by induction of the retrograde response, a pathway that signals mitochondrial stress to the nucleus (Jazwinski and Kriete, 2012).

Among the molecular loci driving replicative ageing is rDNA instability. This results in the formation of extrachromosomal rDNA circles (ERCs), which are retained by the mother cell upon division. Whether the resulting shortened lifespan is due to ERCs themselves or rather to rDNA instability in general remains a matter of discussion. At least two players seem to be involved: the sirtuin Sir2p that suppresses, and the nucleolar protein Fob1p that promotes, homologous recombination in the rDNA. Accordingly, deletion of *FOB1* partially suppresses the short lifespan of *SIR2*-deficient yeast (Kaeberlein *et al*., 1999). Additionally, Sir2p holds further functions associated to replicative ageing: for instance, it seems to modulate the asymmetrical inheritance of oxidatively damaged cytoplasmic proteins (Aguilaniu *et al*., 2003) and, as a histone deacetylase, to be epigenetically active at telomeric sites (Dang *et al*., 2009).

The modulation of growth, metabolism and stress resistance through the nutrient-responsive Ras–PKA and TOR–Sch9 pathways is intimately connected to the regulation of replicative ageing: mutations or regimens (e.g. caloric restriction) that compromise Ras–PKA or TOR–Sch9 signalling extend replicative lifespan (Fabrizio *et al*., 2004a, 2004b; Kaeberlein *et al*., 2005). The corresponding downstream effectors relevant for replicative ageing need further exploration but at least include mRNA translation, stress-responsive transcription factors and mitochondria (Delaney *et al*., 2013; Medvedik *et al*., 2007; Steffen *et al*., 2008). Interestingly, nutrient-responsive signalling seems to converge with other replicative ageing-related pathways on similar downstream events; for instance, the retrograde response is connected to lifespan extension via reduced Ras–PKA signalling (Kirchman *et al*., 1999) and reduced TOR signalling is accompanied by increased rDNA stability (Ha and Huh, 2011; Medvedik *et al*., 2007).

## Ageing in the population: nutrient availability and chronological lifespan

The second ageing paradigm in yeast, the chronological lifespan, represents the time a culture survives in the postdiauxic and stationary phases. Yeast chronological ageing is commonly applied as a model to study factors that impact the ageing of postmitotic tissues and cell types in higher eukaryotes, such as muscle or brain. During yeast chronological ageing, damaged cells challenged with restricted nutrient availability undergo PCD for the benefit of the fitter cells in the population. This not only eliminates unfit individuals that would otherwise consume the scarce resources but also results in the release of nutrients and substances into the surroundings that stimulate the survival of younger cells (Herker *et al*., 2004). Interestingly, stationary yeast cultures differentiate into a quiescent and a non-quiescent subpopulation, the first composed of healthy, unbudded daughter cells with full replicative potential and the second formed by replicatively older cells with higher levels of ROS and markers of apoptosis and necrosis (Allen *et al*., 2006). Importantly, early death during ageing combined with a high mutation frequency, all linked to superoxide as a regulator (Fabrizio *et al*., 2004a, 2004b), seems to favour the adaptation to and growth in a constantly changing environment (adaptive regrowth), thus benefitting the survival of the clonal subpopulation. Even though apoptosis plays a central role in this process (Fabrizio *et al*., 2004a, 2004b; Herker *et al*., 2004), recent evidence suggests that programmed necrosis also represents a crucial cell death type occurring with ongoing chronological age (Carmona-Gutiérrez *et al*., 2011; Eisenberg *et al*., 2009).

Chronological ageing is regulated by the two nutrient-sensing pathways controlled by Ras–PKA and TOR–Sch9. Genetic or nutrient-dependent downregulation of these pathways (e.g. through caloric restriction) stimulates the protein kinase Rim15p, which in turn promotes the activation of the stress-response transcription factors Msn2/4p and Gis1p, finally resulting in extended chronological lifespan (Pedruzzi *et al*., 2003). Of note, both nutrient-dependent pathways also modulate replicative ageing (see above), reflective of a connection between both ageing models. In fact, the replicative potential of chronologically aged mother cells is decreased (Ashrafi *et al*., 1999). However, this coupling is intricate, as evinced by Sir2p, which is associated to both ageing models but with partly opposed outcomes. Indeed, deletion of *SIR2* shortens replicative lifespan (see above) but has no effect on chronological lifespan under standard conditions, or even extends it under caloric restriction (Fabrizio *et al*., 2005; Smith *et al*., 2007). In addition, crucial metabolites such as acetyl-CoA or polyamines (Carmona-Gutiérrez *et al*., 2011; Eisenberg *et al*., 2009, 2014), and the modulation of key homeostatic elements such as vacuolar pH (Ruckenstuhl *et al*., 2014), are increasingly being acknowledged as pivotal regulators of chronological ageing. It should be noted that a controversy exists about the impact of acetic acid (a common end-product of alcoholic fermentation) as an extrinsic stress on chronological ageing (Ludovico *et al*., 2001; Burtner *et al*., 2009; Weinberger *et al*., 2010; Giannattasio *et al*., 2005). One view supports the idea that extrinsic acetic acid drives media acidification and is the primary cause of yeast chronological ageing, while the other view argues that acetic acid and media acidification are separate and non-exclusive pro-ageing factors (Longo *et al*., 2012). Irrespectively, this model has demonstrated to not just reflect ‘private’ mechanisms specific for yeast chronological ageing but to be exceptionally effective in the elucidation of general senescence processes.

The complex nature of chronological ageing is mirrored by its manifold downstream effects, among them mutagenesis and replication stress (Fabrizio *et al*., 2005; Weinberger *et al*., 2007), oxidative stress (Fabrizio *et al*., 2003; Herker *et al*., 2004), mitochondrial dysfunction (Bonawitz *et al*., 2006; Scheckhuber *et al*., 2011), lipid changes (Beach *et al*., 2013; Eisenberg and Büttner, 2013) or a reduction in cellular ‘self-recycling’ (autophagy) (Eisenberg *et al*., 2009; Yorimitsu *et al*., 2007). For instance, the polyamine spermidine extends chronological lifespan by induction of autophagy, ultimately leading to the inhibition of programmed necrosis. Intriguingly, this induction is regulated by the deacetylation of histone H3 (Eisenberg *et al*., 2009). In fact, mounting evidence ascribes epigenetic cell death regulation a determinant role during chronological ageing (Carmona-Gutiérrez *et al*., 2011; Eisenberg *et al*., 2009; Schroeder *et al*., 2013). In accordance with a vast regulatory interplay, molecular factors from virtually all cellular locations have been implicated in the regulation of chronological ageing, including mitochondrial, nuclear, vacuolar, peroxisomal and cytoplasmic effectors.

## Ageing in a colony: differentiation through region-specific cell death

The proliferation and ageing of *S. cerevisiae* in its natural habitat mainly happens in the form of colony formation on solid surfaces, such as fruits and vegetables, a life style that hardly resembles the growth in liquid medium common in laboratory conditions. The growth of a single yeast colony can be divided into a phase of rapid growth, in which the cells genetically and biochemically largely resemble cells dividing in liquid culture, and a subsequent phase of slower growth, in which the colony starts to diversify. Cells at the colony periphery proliferate, while the cells in the colony centre enter stationary phase (Meunier and Choder, 1999). During growth and ageing, these colonies undergo distinct developmental phases, thereby periodically changing the pH of their surroundings from acidic to alkaline and vice versa and generating various metabolite and nutrient gradients around the colony via consumption, secretion and diffusion. A first alkaline phase starts early and lasts about 1–2 days, after which colonies enter the slow-growth phase and start to acidify their surroundings. The second alkaline phase starts at around day 8–10 and is characterized by massive metabolic changes. Thereby, an ammonia signal controlled by the transcriptional regulator Sok2p governs differentiation and metabolic reprogramming within the distinct subpopulations of the colony (Palková *et al*., 1997). Importantly, the induced metabolic changes, which include a repression of genes regulating mitochondrial respiration and stress response as well as an upregulation of genes involved in fatty acid oxidation, amino acid metabolism and peroxisome biogenesis, are restricted to the outer regions of the colony and promote long-term survival (Palková *et al*., 2002). Furthermore, expression of plasma membrane transporters specific for ammonia and for carboxylic acids (by-products of early colony development, such as pyruvate, acetate or lactate), as well as of additional transporters that contribute to pH alterations, is elevated in the switch from acidic to alkaline (Paiva *et al*., 2013; Palková *et al*., 2002). The uptake of carboxylic acids (by Jen1p) is coupled with proton uptake, leading to an increase in extracellular pH that might be essential for the alkaline phase and the connected secretion of ammonia. Jen1p, which is specifically upregulated in the outer regions, is essentially involved in colony differentiation, as its absence leads to insufficient ammonia production and thus incapability to transit into the alkaline phase (Paiva *et al*., 2013).

In the colony centre, the metabolism remains mostly unaltered. Cells exhibit accumulating oxidative stress and undergo PCD with typical apoptotic markers (Váchová and Palková, 2005). Importantly, removal of the old cells in the colony centre inhibited further growth at the colony margin, indicating that PCD in the centre region acts as crucial factor in promoting longevity of the colony (Váchová and Palková, 2005). In contrast to mere cell lysis, such PCD activation prevents the release of potentially harmful lytic enzymes or additional toxic components that might damage still healthy surrounding cells. Thus, apoptotic death of centrally located cells might serve a purpose similar to the apoptotic death of individual cells in metazoan organisms. While Sok2p has been identified as an essential mediator of colony differentiation through region-specific cell death regulation, the molecular pathways executing apoptosis of centre cells remain mostly elusive. Two apoptotic key players, Yca1p and Aif1p, as well as cytosolic Sod1p, which has been shown to be pivotal for yeast longevity during chronological ageing, are all dispensable for region-specific death and concomitant long-term survival of colonies (Cáp *et al*., 2009; Váchová and Palková, 2005). However, Ras–cAMP–PKA signalling, which functions in apoptosis and ageing, seems to be involved in the differentiation into death zones within a yeast colony in a manner dependent on *WHI2* (Leadsham *et al*., 2009).

Besides the differentiation into central and outer cellular sectors, the aged cells in the centre form two distinct subpopulations, starting after about 7 days. Within the upper region, cells exhibit high stress resistance and an extended lifespan, while cells in the lower layers are sensitive to stress and die fast (Cáp *et al*., 2012). Typical characteristics of cells from the upper layer (still healthy, aged cells), such as activated glycolysis and inhibited mitochondrial respiration, resemble the metabolic profile of typical long-lived mutants such as Δ*tor1*, Δ*sch9* and Δ*ras2* during chronological ageing in liquid culture, suggesting that these might represent typical traits for longevity. Highlighting the impact of mitochondrial respiration on colony development, respiratory deficiency has been shown to suppress the accumulation of ROS and apoptosis in yeast colonies (Ruckenstuhl *et al*., 2009). Accordingly, forced respiration during seeding of a yeast colony inhibited the formation of a colony, an effect that could be ameliorated via supplementation with the ROS scavenger glutathione. These data indicate that during the initial phases of colony development, high levels of glycolysis and especially a repression of respiration are favourable, which is reminiscent of the Warburg effect in cancer cells. This suggests that the development and ageing of yeast colonies can be efficiently used to study (at least some) features of tumour development in higher eukaryotes, including metabolic traits, such as glycolysis and oxidative phosphorylation. Interestingly, the isolated growth of yeast colonies derived from single cells has been hypothesized to model distinct phenotypes of human ageing, such as the age-dependent formation of skin lesions called ‘senile warts’. These isolated colonies started to generate little warts with progressing age, which contained cells that display increased viability and a higher mutation frequency as compared to cells from the smooth layer of the same colony (Mazzoni *et al*., 2012). This implies that the ageing of a differentiated yeast colony can be used not only to study microbial communication, stress defence, adaptation, programmed cell death and ageing in defined regions of a structured ‘multicellular organism’ but also to model more specific phenotypes of human ageing.

## Concluding remarks

Yeast ageing is a complex process involving multiple pathways that are engaged as a response to the manifold physiological situations encountered in nature. As a general phenomenon on which all ageing subtypes converge, PCD seems to be the physiological event that, by regulating cellular lifespan, controls population dynamics. By this altruistic death of single cells that eliminates damaged individuals, the clonal community ensures the possibility of adapting to the changing environment, thus promoting its long-term survival. This suggests that ageing might, at least to some extent, hold programmatic features as an adaptive response to cope with ever rearranging and challenging conditions. Necessarily, this idea leads to controversial group selection theories (Blagosklonny, 2013; Longo *et al*., 2005; Gonidakis and Longo, 2009), by which groups (of unicellular or multicellular organisms) would have an evolutionary advantage if they retained the capability to coordinate a regulated clearance of unfit individuals. Indeed, during chronological ageing, for instance, diminishing the capacity of a yeast population to undergo apoptosis results in a competitive disadvantage towards a death-competent population over time (Herker *et al*., 2004; Fabrizio *et al*., 2004a, 2004b). This phenomenon is recapitulated in higher eukaryotes, for example in the accelerated ageing of annual plants or Pacific salmon after their flowering or spawning, respectively. These observations, however, do not exclude that other non-programmatic and rather stochastic elements suggested in alternative ageing theories co-determine the ageing process, such as cumulative damage incurred by hazardous oxygen radicals over time, as implicated in the ‘free radical theory of ageing’. In any case, cells eventually succumb to ageing. While apoptosis was first identified as the PCD form managing these lethal events, mounting evidence suggests that other subtypes might be involved in ageing regulation. Programmed necrosis, for instance, seems to be pivotal in the chronological ageing model, and it will be interesting to explore how necrotic events influence colony differentiation and whether execution via a specific cell death type underlies a guided physiological purpose, for instance in intercellular communication.

**Figure 2:**
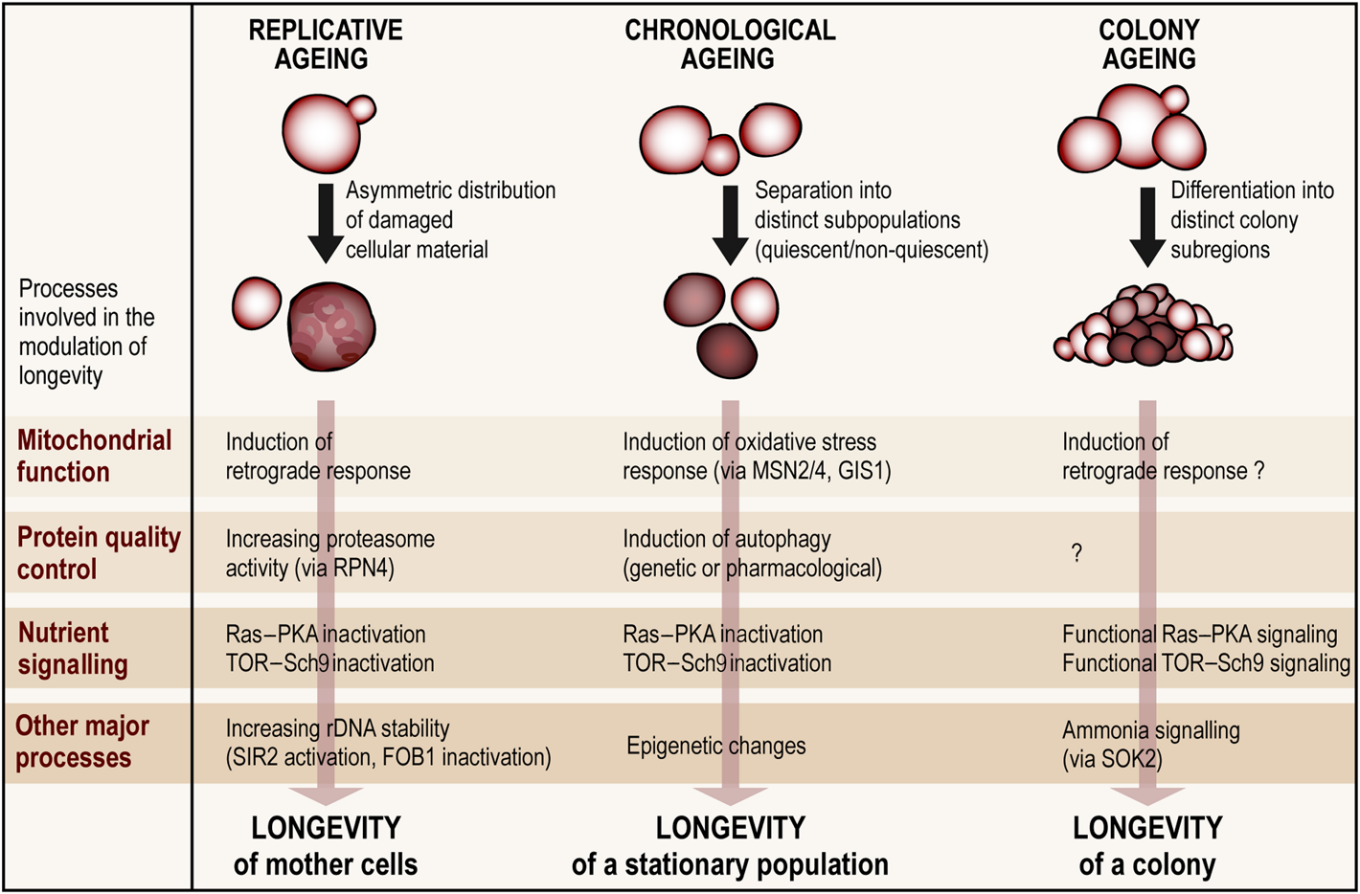
Processes involved in the regulation of replicative, chronological and colony ageing. Different molecular processes govern the modulation of yeast longevity, among them processes involved in mitochondrial function, protein quality control and nutrient signalling. Some of the corresponding mechanisms in each ageing model remain to be elucidated; some are specific for a given paradigm, while others either completely or partly overlap. Several mechanisms even result in opposed outcomes: the inactivation of main nutrient signalling pathways (Ras/PKA and TOR–Sch9), for instance, causes replicative and chronological longevity but results in the demise of a colony in the long run, since the region-specific death in its centre is necessary for colony differentiation. Note that the mechanisms shown in the figure represent only examples for each process and do not constitute the entirety of known pathways for each ageing model

Importantly, the environmental conditions dictate whether either proliferation, stationary behaviour or colony formation and differentiation is sustained, thus defining the distinct ageing paradigms. Thus, some regulatory and executing elements do not overlap while others do (Figure 2). For example, Yca1p and Aif1p are involved in apoptotic death during chronological but not during colony ageing. Instead, the Ras–cAMP pathway plays a lethal role in all ageing models. Moreover, in liquid culture, longevity during stationary phase appears to rely on the mutation rate frequency to generate appropriately adapted genetic variants, as well as on early PCD of (replicatively) older cells to spare and generate nutrients. Instead, in differentiated colonies, mutation frequencies rather play a subordinate role and long-term survival is governed by adaptive events, at least in part mediated by Sok2p and ammonia signalling. Another example is that both a chronologically aged population and one aged within a colony on solid ground divide into differently fit subpopulations. However, the characteristics of these subpopulations partly differ: in an aged colony, the fitter cells at the periphery still slowly proliferate, while the healthier fraction of a stationary phase liquid culture is composed of quiescent virgin cells. At the transcriptional level, genes necessary for fatty acid oxidation and carboxylic acid uptake or metabolism are upregulated in both of these subpopulations. Instead, transcripts involved in oxidative stress defence are increased in quiescent stationary cells, but decreased in cells of the colony's outer regions. This variation might be due to the fact that, in contrast to cells in liquid culture, cells within a colony have distinct positions, neighbours and access to nutrients. Given these similarities and disparities, it is important to assess where the regulatory network controlling ageing under different conditions interconnects. Here, mitochondrial and nutrient signalling pathways, which are crucially involved in all ageing models, are good candidates to be such a molecular hub. For instance, it will be exciting to explore whether autophagy, whose paramount significance for chronological ageing is being unveiled, and whose activity is directly linked to nutrient availability, is also as determinant for other ageing types.

Besides the ageing subtypes observed under laboratory conditions, there might be more and/or their relevance might be relative in the wild. For instance, the apoptotic death of replicatively old mother cells, which do only represent a very minor percentage of the population, can hardly contribute to the fitness of a clone. As old mothers are very scarce, their death neither spares nor releases notable nutrients. Thus, the apoptotic death of replicatively old mother cells most probably does not represent an adaptive trait, but might solely represent the consequence of accumulating cellular damage. Under natural conditions, the portion of cells achieving a replicatively old status is probably even lower. In stationary phase, the biggest fraction will rather be composed of virgin cells, or cells that have undergone several divisions only. However, physiological changes related to replicative ageing that affect, for example, certain cellular stress resistances already occur after only four or five divisions (Sorokin *et al*., 2014). While most laboratory yeast strains form smooth colonies, natural yeast isolates rather build structured biofilm colonies that differ in architecture and the distribution of subpopulations. These structured colonies form a layer of stationary-phase cells characterized by high stress resistance on top of the colony, while cells in the interior keep proliferating, where they are protected against environmental threats and effects. In domesticated laboratory strains this trait might have been lost, due to invariably harmless conditions and therefore a lack of evolutionary pressure. Thus, ageing in a biofilm colony might represent yet another ageing subtype. As we keep on deciphering how yeast cells and populations age, we'll continue learning from old yeast for ourselves.
